# ASSOCIATION OF CREATININE- AND CYSTATIN-C–BASED INDICES WITH INCIDENT SARCOPENIA: A LONGITUDINAL STUDY

**DOI:** 10.1093/geroni/igae098.3801

**Published:** 2024-12-31

**Authors:** Jae Young Jang, Hyung Eun Shin, Daehyun Lee, Heeeun Jung, Hyunjin Cho, Nahyun Lim, Chang Won Won, Miji Kim

**Affiliations:** Kyung Hee University, Seoul, Republic of Korea; Kyung Hee University, Seoul, Republic of Korea; Kyung Hee University, Seoul, Republic of Korea; Kyung Hee University, Seoul, Republic of Korea; Kyung Hee University, Seoul, Republic of Korea; Kyung Hee University, Seoul, Republic of Korea; Kyung Hee University, Seoul, Republic of Korea; Kyung Hee University, Seoul, Republic of Korea

## Abstract

Creatinine and cystatin-C–based indices have been reported to be associated with sarcopenia; however, the long-term relationship between these indices and sarcopenia remains unclear. This study examined the longitudinal relationship between baseline serum creatinine and cystatin-C–based indices and incidence of sarcopenia during a 6-year follow-up to compare which indicator well predicts sarcopenia in community-dwelling older adults. This study included 698 participants (mean age=75.1±3.7years; women=54.4%) from the Korean Frailty and Aging Cohort Study (KFACS). Baseline Creatinine-to-cystatin-C ratio estimated glomerular filtration rate (eGFR) ratio (eGFRcystatin-C[mL/min/1.73m2]/eGFRcreatinine[mL/min/1.73m2]), sarcopenia-index (serum creatinine[mg/dL]×eGFRcystatin-C[mL/min/1.73m2]), predicted skeletal muscle mass index (pSMI), and total-body muscle mass index (TBMM) were measured. Sarcopenia was defined by following the guidance of the Asian Working Group for Sarcopenia 2019. The incidence of sarcopenia during 6-year follow-up was 22.5% in men and 16.7% in women. The pSMI and TBMM showed higher area under the receiver operating characteristic curves for predicting the incidence of sarcopenia in men (pSMI==0.634; TBMM=0.635) and women (pSMI=0.727; TBMM=0.724) than other indices. Higher pSMI and TBMM were associated with the incidence of sarcopenia in both men (pSMI, odds ratio [OR]=0.370, 95% confidence interval [CI]=0.223–0.614; TBMM, OR=0.928, CI=0.896–0.962) and women (pSMI, OR=0.149, CI=0.072–0.307; TBMM, OR=0.896, CI=0.857–0.936), after adjusting confounders. pSMI and TBMM showed higher predictive capabilities for the incidence of sarcopenia compared to other indices. Also, higher pSMI was strongly associated with a lower risk of incident sarcopenia. These findings suggest that monitoring pSMI could help predict the onset of sarcopenia in community-dwelling older adults.

